# Antioxidant biomaterials in intervertebral disc regeneration: current status and future clinical translation

**DOI:** 10.3389/fbioe.2026.1854242

**Published:** 2026-06-04

**Authors:** Ziyu Zhang, Runjia Hua, Yudong Wang, Jianing Wang, Xuefeng Li, Feng Zhou, Jian Mi, Li Chu, Chao Jiang, Yanjun Che, Feng Han, Genglei Chu

**Affiliations:** 1 Department of Orthopaedic Surgery, The First Affiliated Hospital of Soochow University, Suzhou, Jiangsu, China; 2 Suzhou Medical College, Soochow University, Suzhou, Jiangsu, China; 3 Yong Loo Lin School of Medicine, National University of Singapore, Singapore, Singapore; 4 Department of Orthopaedics, The Affiliated Suzhou Hospital of Nanjing Medical University, Suzhou Municipal Hospital, Suzhou, Jiangsu, China; 5 Gusu School of Nanjing Medical University, Suzhou, Jiangsu, China; 6 Division of Spine Surgery, Department of Orthopedic Surgery, Nanjing Drum Tower Hospital, The Affiliated Hospital of Nanjing University Medical School, Nanjing, Jiangsu, China

**Keywords:** antioxidant, antioxidant materials, intervertebral disc degeneration, multifunctional composite materials, reactive oxygen species, regenerative therapy

## Abstract

Intervertebral disc degeneration (IVDD) is a leading cause of chronic back pain and long-term disability, imposing a substantial burden on healthcare systems worldwide. At the core of this degenerative process lies oxidative stress, a pathological condition driven by excessive accumulation of reactive oxygen species (ROS). This redox imbalance initiates a destructive cascade within the intervertebral disc, resulting in injury, premature senescence, and death of nucleus pulposus cells. The loss of these critical cells subsequently provokes inflammatory responses and degradation of the extracellular matrix, ultimately compromising the structural integrity of the disc. Although conventional clinical interventions effectively alleviate symptoms, they typically fail to target the underlying biological mechanisms or halt disease progression. This unmet therapeutic need has spurred growing interest in antioxidant materials, which offer a more proactive approach by directly neutralizing ROS and restoring redox homeostasis. Beyond mere ROS scavenging, these materials also mitigate inflammation and foster a microenvironment supportive of tissue regeneration. Current research efforts are increasingly focused on the rational design of such antioxidant systems, particularly their integration with advanced cell-based therapies to enhance regenerative outcomes. A detailed assessment of how these materials modulate specific pathological pathways is essential to clarify their practical utility and inherent limitations. Translating promising laboratory results into clinical practice remains a major challenge, necessitating rigorous evaluation of their performance in complex biological settings. Refining these antioxidant strategies may ultimately pave the way for a paradigm shift, from symptomatic relief to genuine functional restoration in patients with IVDD.

## 1 Introduction

Intervertebral disc degeneration (IVDD) is a progressive disorder widely recognized as a primary contributor to chronic low back pain and various neurological conditions, arising from a confluence of complex and interrelated factors (van Uden et al., 2017; Xin et al., 2022; Wang et al., 2023b). With the global population aging, the prevalence of IVDD continues to rise, leading to considerable patient morbidity and imposing significant socioeconomic burdens (Fine et al., 2023; Ma et al., 2025). As a key structural component of the spine, the intervertebral disc (IVD) comprises a gelatinous nucleus pulposus (NP) at its center, encased by the fibrous annulus fibrosus (AF). This architecture is completed by cartilaginous endplates (CEP), which cap the superior and inferior surfaces of the disc (Sudo, 2024). Notably, the IVD is the largest avascular structure in the human body; this intrinsic lack of blood supply not only restricts nutrient exchange but also fundamentally limits the disc’s capacity for self-repair and redox homeostasis once the oxidative balance is disrupted. In its healthy state, the IVD demonstrates exceptional biomechanical properties, enabling it to effectively absorb and distribute mechanical loads across the spine. However, under the cumulative influence of aging, mechanical stress, genetic predisposition, and inflammatory processes, the disc progressively undergoes structural and functional deterioration (Silwal et al., 2023; Wang et al., 2023a; Xu et al., 2023). Characterized by a loss of nucleus pulposus cells and degradation of the extracellular matrix (ECM), often accompanied by annular tears and elevated inflammation, this degenerative cascade fundamentally compromises the disc’s structural integrity. Over time, these changes result in diminished disc height and reduced elasticity, frequently culminating in clinical complications such as disc herniation and spinal stenosis (Chen et al., 2024a).

Current management of IVDD relies primarily on conservative therapies and surgical interventions, including open fenestration discectomy and the administration of nonsteroidal anti-inflammatory drugs (NSAIDs) (Shah et al., 2017; Hamawandi et al., 2020; Wu et al., 2020). Suboptimal patient adherence to or engagement in rehabilitation programs, as well as the use of nonstandardized techniques by rehabilitation practitioners, can negatively impact treatment outcomes. Moreover, while traditional Chinese medicine approaches are sometimes employed, their efficacy remains limited by a lack of rigorous scientific validation and standardized protocols, rendering results highly variable across individuals. Critically, existing strategies predominantly aim at alleviating symptoms rather than reversing or halting the underlying degenerative process (El Melhat et al., 2024). Surgical options, though sometimes necessary, carry notable drawbacks, including extensive tissue trauma, prolonged recovery periods, and heightened risks of postoperative complications (Chung et al., 2014; Støttrup et al., 2019; Wu et al., 2020). Consequently, there is an urgent need to develop innovative therapeutic approaches that directly target the core pathological mechanisms of IVDD and actively promote disc regeneration.

In recent years, accumulating evidence has demonstrated that oxidative stress (OS) plays a pivotal role in the onset and progression of IVDD (Wang et al., 2023b; Chen et al., 2024e; Zhou et al., 2024c). The intervertebral disc, particularly the nucleus pulposus, resides in a hypoxic, nutrient-poor microenvironment, rendering its cells exceptionally susceptible to oxidative damage. Under conditions of excessive reactive oxygen species (ROS) production coupled with impaired antioxidant defenses, nucleus pulposus cells (NPCs) experience substantial DNA damage, protein oxidation, and lipid peroxidation. These molecular insults subsequently trigger apoptosis, cellular senescence, inflammatory responses, and dysregulated degradation of the extracellular matrix, collectively accelerating disc degeneration (Zhu et al., 2023b; Bu et al., 2024). Given the central involvement of oxidative stress in IVDD pathogenesis, therapeutic strategies that scavenge excess ROS and restore redox homeostasis have gained prominence as promising avenues for intervention.

This review examines recent advances in the application of antioxidant materials for the treatment of IVDD. We first outline the pathophysiological mechanisms by which oxidative stress contributes to disc degeneration. We then provide a comprehensive overview of various antioxidant material platforms, including hydrogels, nanomaterials, and natural antioxidants, detailing their therapeutic applications, strengths, and current limitations. Furthermore, we discuss emerging strategies that integrate these materials with advanced biotherapies such as cell-based and gene therapies, as well as innovations in smart, stimuli-responsive delivery systems. Finally, we synthesize key insights from recent studies, highlight persistent challenges in the field, and propose future directions for the development and clinical translation of antioxidant-based interventions for IVDD. By consolidating current knowledge and identifying critical gaps, this review aims to provide both a theoretical framework and practical guidance to support the design of more precise and effective therapies for intervertebral disc degeneration.

## 2 The pathophysiological mechanism of oxidative stress in intervertebral disc degeneration

IVDD is a complex, progressive disorder characterized by the gradual loss of structural integrity and functional capacity in disc tissue. Driven by multiple underlying factors, this process entails the progressive disintegration of the disc’s internal architecture, ultimately impairing its ability to sustain normal spinal biomechanics. Rather than resulting from a single acute event, IVDD manifests as a cascade of evolving alterations, often initiated at the cellular level, that progressively erode the tissue’s resilience over time (Fine et al., 2023; Sudo, 2024). Central to this degenerative cascade is the depletion and dysfunction of NPCs, which critically undermines the stability of the disc’s core. As these essential cells deteriorate, the surrounding ECM undergoes extensive degradation and maladaptive remodeling, further compromising the disc’s internal scaffold. This internal collapse frequently propagates outward, inflicting damage on the annulus fibrosus (AF) and contributing to cartilaginous endplates (CEP) sclerosis. Collectively, these changes impair the mechanical integrity of the spinal functional unit (Xin et al., 2022; Wang et al., 2023b). Among the various contributors to IVDD, OS is widely recognized as a pivotal driver that initiates and accelerates disc degeneration through multiple direct and indirect molecular pathways (Chen et al., 2024e; Zhou et al., 2024c).

**FIGURE 1:**
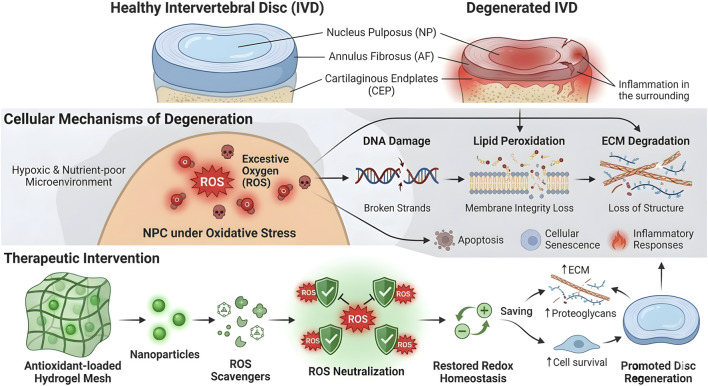
Oxidative stress–driven pathogenesis and therapeutic potential of antioxidant strategies in IVDD. Mechanical stress and aging induce mitochondrial dysfunction in nucleus pulposus cells (NPCs), resulting in the accumulation of reactive oxygen species (ROS). Excess ROS cause DNA damage, protein oxidation, and lipid peroxidation, ultimately triggering NPC apoptosis, senescence, inflammatory responses, and degradation of the ECM. Antioxidant strategies counteract this cascade by neutralizing ROS and restoring redox homeostasis, thereby mitigating the progression of IVDD.

Oxidative stress arises when the delicate equilibrium between the production and clearance of ROS is disrupted, resulting in ROS accumulation that ultimately inflicts damage on cells and tissues (Figure 1). In the degenerating intervertebral disc, multiple factors, including mitochondrial dysfunction, inflammatory responses, and mechanical stress, act as major sources of ROS. These are further amplified by ischemia, hypoxia, and metabolic alterations. NPCs are especially vulnerable to this oxidative burden, as they already reside in an intrinsically harsh microenvironment marked by low oxygen tension and limited nutrient availability. Consequently, excessive ROS accumulation acts as a potent catalyst, initiating a cascade of pathological events that accelerate tissue deterioration (Mai et al., 2025). In diseased discs, the absence of a direct blood supply, compounded by sclerosis of the cartilaginous endplates, severely restricts metabolic exchange, leading to a pronounced buildup of ROS. This metabolic bottleneck impairs the replenishment of endogenous antioxidants, creating a deficit that the avascular disc tissue cannot compensate for. The resulting chronic redox imbalance inflicts severe damage to cellular structures and disrupts essential biological functions, establishing oxidative stress as a central driver in the progression of intervertebral disc degeneration (Rahal et al., 2014; Li et al., 2022). These disruptions promote accelerated apoptosis of nucleus pulposus cells and extensive degradation of the ECM, progressively compromising both the structural integrity and biomechanical function of the disc. Beyond its direct cytotoxic effects, oxidative stress also serves as a powerful amplifier of inflammation, exacerbating the entire degenerative cascade. This pathogenic influence is largely mediated through a network of redox-sensitive signaling pathways, most prominently the nuclear factor erythroid 2-related factor 2 (Nrf2)/Kelch-like ECH-associated protein 1 (Keap1) axis. As the principal endogenous antioxidant defense system in disc cells, the Nrf2/Keap1 pathway represents the most critical mechanism for preserving cellular homeostasis under oxidative challenge within the disc microenvironment (Chen et al., 2024c). Under physiological conditions, Nrf2 is bound to Keap1, which targets it for ubiquitin-mediated proteasomal degradation, thereby maintaining low basal levels of Nrf2 in the cytoplasm. Upon exposure to oxidative stress, critical cysteine residues in Keap1 are modified, inducing a conformational change that impairs its ability to target Nrf2 for ubiquitin-proteasomal degradation. Consequently, newly synthesized Nrf2 escapes Keap1-mediated turnover, stabilizes, accumulates, and translocates into the nucleus, where it activates the transcription of essential downstream antioxidant genes (Chen et al., 2026). This molecular switch enhances cellular defenses by upregulating antioxidant enzymes and promoting glutathione (GSH) synthesis, effectively reinforcing the cell’s intrinsic capacity to neutralize ROS. Such a dynamic adaptive response serves as a critical buffer, significantly augmenting the tissue’s overall antioxidant resilience against environmental stressors (Li Y. et al., 2025; Zhang H. et al., 2025). Numerous natural compounds and therapeutic agents, including atorvastatin, casticin, and resveratrol, have been demonstrated to mitigate IVDD through activation of the Nrf2 pathway (Wu L. et al., 2025). A major advantage of these strategies is their ability to harness the cell’s endogenous defense systems, thereby generating durable protective effects. Nevertheless, the efficacy and specificity of Nrf2 activation can be modulated by factors such as cellular context and drug concentration.

### 2.1 Cellular injury and death

Excessive ROS exert pathological effects by directly damaging critical intracellular biomolecules, particularly DNA, proteins, and lipids. DNA damage can induce deleterious mutations and impair cellular function, while protein oxidation often diminishes enzymatic activity and destabilizes structural components. Lipid peroxidation, in turn, compromises the integrity of cellular membranes, further eroding the cell’s physical barrier. This cumulative molecular damage ultimately activates multiple forms of regulated cell death in NPCs, including apoptosis, necroptosis, and ferroptosis, all of which contribute significantly to the progression of IVDD (Bu et al., 2024; Gao et al., 2024; Huang et al., 2024; Lao et al., 2024; Fu et al., 2025; Song et al., 2025; Zhu et al., 2025). For instance, one study demonstrated that hesperidin attenuates oxidative stress–induced ferroptosis in NPCs via the Nrf2/NF-κB signaling pathway, thereby protecting intervertebral discs from degeneration (Chen J. et al., 2023). Similarly, Sun et al. reported that catalytic nanoparticles suppress necroptosis in NPCs and mitigate oxidative stress–driven IVDD (Sun et al., 2024). Recent evidence highlights ferroptosis as a particularly prominent mechanism in disc degeneration, especially within NPCs. As an iron-dependent form of regulated cell death, ferroptosis is primarily driven by the accumulation of lipid peroxides and ROS, which jointly undermine cellular homeostasis. Unlike classical cell death pathways, ferroptosis stems from the failure of antioxidant systems—most notably the inactivation of glutathione peroxidase 4 (GPX4)—to counteract iron-dependent lipid peroxidation, establishing it as a distinct and critical contributor to disc tissue dysfunction. *In vivo* studies using rat models further support this notion: treatment with ginsenoside Rg3 preserved disc height and slowed the progression of degeneration (Xu et al., 2025). Sirtuin 3 (Sirt3) plays a central role in this context by regulating mitochondrial ROS levels and serving as a key guardian against ferroptosis. Depletion of ubiquitin-specific peptidase 11 (USP11) leads to accelerated degradation of Sirt3, exacerbating oxidative stress and promoting iron-dependent cell death. This disruption of the USP11–Sirt3 axis markedly aggravates the pathological course of IVDD. In preclinical models, disruption of this protective USP11–Sirt3 signaling cascade correlates with more severe degenerative changes and associated pain-related behavioral deficits (Zhu et al., 2023a). Collectively, these findings point toward promising diagnostic and therapeutic avenues aimed at enhancing NPC survival and alleviating pain in IVDD.

### 2.2 Cellular senescence

Cellular senescence is characterized by an irreversible arrest of cell proliferation coupled with the acquisition of a distinct pro-inflammatory secretory phenotype. Far from being inert, senescent cells actively shape their microenvironment by releasing a spectrum of bioactive factors that foster chronic inflammation and promote localized tissue breakdown within the intervertebral disc (Le Maitre et al., 2007). ROS are key inducers of this senescent state. In NPCs, senescence manifests as a marked decline in proliferative capacity alongside heightened secretion of pro-inflammatory cytokines and matrix-degrading enzymes, changes that collectively exacerbate IVDD (Wang et al., 2023b; Chen et al., 2024e; Fan et al., 2024). For example, Bu et al. demonstrated that glutathione-derived carbon dots mitigate NPC senescence and slow IVDD progression by scavenging ROS and restoring intracellular redox homeostasis (Bu et al., 2024). In another study, Zhang et al. reported that glutamine suppresses glycolysis-mediated AMPKα palmitoylation, thereby inhibiting NPC aging, enhancing autophagy, and ultimately ameliorating IVDD (Zhang Y. et al., 2024).

### 2.3 Mitochondrial dysfunction

Mitochondria serve as the primary energy-producing organelles essential for maintaining NPC viability and function. Their dysfunction represents a critical early event in IVDD pathogenesis. Under the harsh microenvironment of a degenerating disc, characterized by hypoxia, nutrient deprivation, and elevated oxidative stress, mitochondria are exposed to excessive ROS, which disrupt the integrity of the inner mitochondrial membrane. This oxidative insult impairs electron transport chain activity and suppresses ATP synthesis, precipitating a cellular energy crisis that directly compromises disc homeostasis (Zhou et al., 2025). This bioenergetic failure severely hampers NPCs’ capacity to synthesize key ECM components, particularly type II collagen and aggrecan. As these structural proteins decline, the disc loses its fundamental building blocks, progressively diminishing its mechanical resilience and ability to withstand physiological loads. Compounding the problem, damaged mitochondria generate additional ROS, establishing a self-amplifying loop known as “ROS-induced ROS release.” Even more consequential is the leakage of mitochondrial DNA (mtDNA) into the cytosol following organelle damage. This misplaced mtDNA is recognized by the cytosolic DNA sensor cyclic GMP–AMP synthase (cGAS), which interprets it as a danger signal. The resulting activation of the stimulator of interferon genes (STING) pathway triggers a robust type I interferon response and sustained inflammation, further accelerating tissue degradation (Wu D. et al., 2025). Notably, Yang et al. demonstrated that targeted modulation of the mtDNA/SPARC–STING signaling axis, achieved through the delivery of engineered exogenous mitochondria endowed with antioxidant and anti-inflammatory properties, effectively attenuates IVDD progression (Yang G. et al., 2025). Together, these findings underscore that mitochondria are not merely passive targets of oxidative injury but active contributors to disc degeneration. By simultaneously acting as both sources and sensors of cellular stress, they bridge metabolic dysfunction and innate immune activation, positioning them as a pivotal therapeutic target for antioxidant-based strategies aimed at halting IVDD.

### 2.4 Endoplasmic reticulum stress

Endoplasmic reticulum stress (ERS) is tightly intertwined with oxidative stress, creating a self-reinforcing cycle of cellular dysfunction. Accumulation of ROS commonly induces ERS, which in turn activates the unfolded protein response (UPR) as the cell strives to restore proteostasis. This adaptive response is mediated by three principal ER-resident sensors: PERK, IRE1α, and ATF6. However, when ERS becomes chronic or overwhelming, the UPR shifts from a protective to a pro-apoptotic signal—largely driven by sustained CHOP expression—ultimately promoting NPC apoptosis and accelerating IVDD. Lu et al. demonstrated that sulforaphane effectively alleviates ERS in nucleus pulposus cells by activating the Nrf2/HO-1 signaling pathway. This intervention restores cellular homeostasis and significantly slows the pathological progression of IVDD (Lu et al., 2023). Similarly, Chen et al. reported that Sal003, a selective inhibitor of eIF2α dephosphorylation, mitigates ERS-mediated apoptosis and attenuates IVDD progression (Chen Y. et al., 2023).

### 2.5 The vicious cycle of oxidative stress–inflammation–matrix degradation–immune response

The core pathophysiology of IVDD centers on a self-perpetuating cascade driven by oxidative stress. At its heart lies a bidirectional interplay between ROS and inflammation. Excessive ROS act as key signaling molecules that activate major pro-inflammatory pathways in disc cells, most prominently NF-κB. This activation converts a redox imbalance into a sustained inflammatory response, accelerating the localized tissue damage characteristic of IVDD (Burt et al., 2024; Liu W. et al., 2024; Wang T. et al., 2024). Once activated, these pathways robustly upregulate the expression and release of inflammatory mediators, including IL-1β, TNF-α, and IL-6. These cytokines are not passive byproducts; they establish a positive feedback loop by stimulating further ROS production and directly initiating ECM degradation (Li Z. et al., 2023). The ECM forms the structural and functional foundation of the disc, and its progressive disintegration is a defining feature of degeneration. ROS drive ECM breakdown through dual mechanisms: direct oxidative damage to matrix components and indirect upregulation of matrix-degrading enzymes, particularly MMPs and ADAMTS, via inflammatory signaling. This multifaceted assault on critical macromolecules such as collagen and proteoglycans ultimately depletes the disc of its elasticity and load-bearing capacity, leading to a profound loss of biomechanical integrity (Wang et al., 2023b; Chen et al., 2024e). As ECM degradation and tissue damage progress, the local microenvironment undergoes substantial remodeling, generating persistent damage signals that trigger the third phase: immune microenvironment dysregulation. This altered milieu recruits and activates immune cells, primarily macrophages (Burt et al., 2024). Within the disc, macrophages exhibit functional plasticity, polarizing into distinct phenotypes. Classically activated M1 macrophages release a broad array of pro-inflammatory factors that amplify inflammation and exacerbate tissue destruction, whereas alternatively activated M2 macrophages attempt to counteract damage by secreting anti-inflammatory and pro-reparative mediators. Critically, the oxidative and inflammatory conditions established in earlier phases dynamically regulate macrophage polarization, forming a complex feedback circuit that profoundly influences the trajectory and severity of degeneration (Koroth et al., 2023a; Ribeiro-Machado et al., 2023; Ren et al., 2025). M1 macrophages undergo a robust respiratory burst mediated by NADPH oxidase (NOX) on their surface, releasing substantial amounts of superoxide anions and thereby establishing a feedback loop that links inflammation/immunity to oxidative stress (Cameron et al., 2019; Casey et al., 2025).This dynamic is exemplified by work from Fan et al., who demonstrated that exosomes derived from M1 macrophages actively promote IVDD. By activating the LCN2/NF-κB signaling axis, these extracellular vesicles function as potent inflammatory messengers that induce NPC senescence and accelerate overall tissue decline (Fan et al., 2024).

**FIGURE 2:**
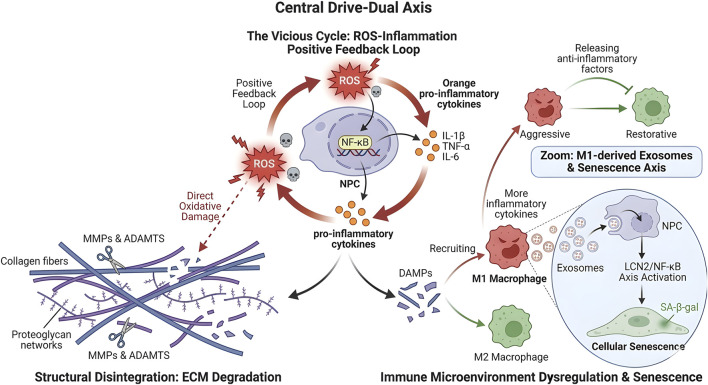
The malignant self-reinforcing network of IVDD driven by oxidative stress and inflammation. Excessive ROS initiates a pathological cascade in IVD cells through NF-κB signaling, inducing the secretion of pro-inflammatory cytokines (IL-1β, TNF-α, IL-6) and proteolytic enzymes (MMPs/ADAMTSs). The resulting ECM degradation releases DAMPs, which feed back to amplify oxidative stress. Concurrently, ROS promotes M1 macrophage polarization, and exosomes from these cells deliver LCN2 to further activate NF-κB, thereby driving cellular senescence and the SASP. This interconnected network ultimately leads to biomechanical collapse, structural failure, and chronic pain during IVDD progression.

Ultimately, oxidative stress acts as a central driver in the pathophysiology of IVDD. It simultaneously triggers cell death, cellular senescence, and inflammatory responses while disrupting mitochondrial and endoplasmic reticulum homeostasis, collectively orchestrating widespread ECM breakdown. This multifaceted dysfunction overwhelms the disc’s intrinsic repair capacity and accelerates degeneration (Figure 2). Given the limited efficacy of current IVDD therapies and gaps in understanding the role of oxidative stress in disease mechanisms, antioxidant biomaterials have emerged as a promising therapeutic approach. Compared with conventional conservative treatments, they offer more sustained effects and fewer adverse reactions (Liu et al., 2023). Therefore, developing antioxidant-based strategies that effectively scavenge ROS and restore redox balance remains critical to delaying, or potentially reversing, IVDD progression. By targeting these upstream oxidative drivers, such interventions stabilize the cellular microenvironment and hold significant potential for preserving the disc’s structural and functional integrity over time.

## 3 Conventional antioxidant strategies and their limitations

Various natural plant extracts, including catechins, flavonoids, and polyphenolic compounds, can neutralize free radicals and other reactive oxidizing species. By directly scavenging these harmful molecules, these bioactive agents act as potent cellular defenders, demonstrating significant mitochondrial protective and antioxidant effects that help preserve cellular homeostasis (Zhou et al., 2024c). In addition, they possess anti-inflammatory properties that alleviate mitochondrial damage and oxidative stress exacerbated by inflammatory responses (Kajarabille and Latunde-Dada, 2019). A thorough assessment of these conventional antioxidant approaches, including their practical applications and inherent limitations in treating intervertebral disc degeneration, is essential before advancing to biomaterial-based strategies. Early research in this area focused primarily on using natural or synthetic antioxidants to counteract the oxidative stress that fuels disc degeneration by neutralizing ROS. Similarly, synthetic small-molecule antioxidants such as N-acetylcysteine (NAC) have shown promise in preclinical IVDD models, yet they encounter identical barriers related to rapid renal clearance and insufficient disc penetration (Wu et al., 2023).

Natural products offer several advantages, such as wide availability, low cost, and a well-established safety profile from long-term dietary consumption. Plant-derived compounds like resveratrol, curcumin, and quercetin modulate the pathological progression of IVDD through multiple biological mechanisms. Beyond scavenging ROS to shield nucleus pulposus cells from oxidative injury, these agents suppress key inflammatory pathways, particularly NF-κB. This dual action downregulates the expression of pro-inflammatory mediators, specifically TNF-α, IL-1β, and IL-6, thereby attenuating the chronic inflammatory milieu within the disc. Although *in vitro* and animal studies confirm that these natural compounds can delay structural deterioration and improve functional outcomes, providing a robust theoretical basis for novel therapies, their clinical translation remains limited by substantial practical barriers (Alharbi et al., 2025; Li X.-L. et al., 2025). A major challenge is their poor *in vivo* stability: they are often rapidly metabolized or degraded, resulting in low bioavailability and difficulty achieving therapeutic concentrations at the target site (Chen et al., 2024e; Wang Y. et al., 2025). Moreover, systemic administration typically fails to deliver these agents selectively to disc tissue, potentially causing off-target side effects and reducing drug accumulation in the affected intervertebral disc (Wang X. et al., 2025). The disc’s unique anatomy, characterized by its avascular nature and sparse cellularity, further impedes effective drug penetration and retention (Zhang C. et al., 2025).

### 3.1 Flavonoids

Flavonoids represent a diverse class of plant polyphenols with well-characterized antioxidant properties. Among these, the isoflavone glycitin has demonstrated significant therapeutic potential in IVDD. Glycitin is a prominent natural isoflavone with significant therapeutic potential. As demonstrated by Zhao et al., it effectively inhibits the NF-κB pathway, thereby attenuating TNF-α–induced metabolic dysregulation and extracellular matrix degradation in nucleus pulposus cells. By dampening these inflammatory signals, glycitin also reduces oxidative stress and mitochondrial damage, helping to preserve the cellular environment against key features of disc degeneration (Zhao et al., 2023). *In vivo* studies further confirm its efficacy in alleviating degenerative phenotypes associated with IVDD. Similarly, Zhu et al. reported that hesperidin, a potent flavonoid, protects the intervertebral disc by inhibiting oxidative stress–induced ferroptosis. Through modulation of the Nrf2/NF-κB signaling axis, hesperidin sustains nucleus pulposus cell survival and prevents the iron-dependent cell death that typically accelerates tissue breakdown (Zhu et al., 2023b). Myricetin has also been shown to partially protect rat intervertebral discs from degeneration via the Nrf2/HO-1/NF-κB signaling pathway (Mao and Fan, 2024). Collectively, these findings underscore the potential of natural flavonoids as multitargeted agents for IVDD treatment. Nevertheless, such small-molecule antioxidants often exhibit short half-lives *in vivo*, necessitating frequent dosing to maintain therapeutic concentrations (Mai et al., 2025). This repeated administration frequently compromises patient compliance and increases the risk of systemic toxicity. Given that IVDD involves a complex network of overlapping pathological processes, interventions relying on a single antioxidant mechanism are rarely sufficient to halt or reverse disease progression. This inherent complexity underscores the limitations of conventional monotherapies in addressing the multifaceted nature of disc degeneration.

### 3.2 Isothiocyanates and endogenous antioxidant modulation

Sulforaphane (SFN), a potent Nrf2 agonist, effectively alleviates endoplasmic reticulum stress in NPCs by activating the Nrf2/HO-1 signaling pathway. This mechanism restores cellular homeostasis and reduces molecular damage, significantly delaying IVDD progression (Lu et al., 2023). *In vitro* experiments confirm that SFN upregulates HO-1 expression and thereby protects NPCs. These findings are further supported by *in vivo* evidence showing that SFN treatment reduces endoplasmic reticulum stress and slows disc degeneration in rat models. Moreover, recent data indicate that LAMP1-mediated lipophagy plays a critical role in suppressing NP cell senescence, a process that sulforaphane activates to decelerate IVDD progression (Qin et al., 2025). By simultaneously targeting lipid accumulation and cellular senescence, sulforaphane represents a highly effective approach to delaying intervertebral disc degeneration (IVDD). Such dual action on underlying metabolic and aging pathways provides a promising foundation for future clinical strategies aimed at preserving disc health and longevity. However, achieving therapeutic effects with high-dose antioxidants may increase the risk of toxicity. For example, GSH, a key endogenous antioxidant, has been extensively studied in the context of IVDD (Li F. et al., 2024). Although modulating GSH levels can markedly influence ECM integrity and cell survival, safely and effectively elevating GSH concentrations within the disc remains challenging.

### 3.3 Alkaloids

Alkaloids, a diverse group of naturally occurring nitrogenous compounds, have gained significant attention for their potent antioxidant and anti-inflammatory properties in intervertebral disc repair.Among these, evodiamine, a major bioactive alkaloid, has been shown to ameliorate IVDD by modulating the Nrf2 and MAPK pathways (Xie et al., 2024). It protects NPCs by inhibiting mitochondrial dysfunction, ECM degradation, and inflammatory responses. In particular, evodiamine counteracts TBHP-induced mitochondrial impairment in NPCs, thereby preventing ECM breakdown and dampening the inflammatory milieu. This dual action alleviates IVDD in puncture-induced rat models. Mechanistically, these effects stem from concurrent activation of the Nrf2 pathway and suppression of the MAPK pathway in NPCs. Ultimately, evodiamine attenuates IVDD progression through coordinated modulation of the Nrf2/HO-1 and MAPK signaling axes, mounting a multifaceted defense against cellular and structural decay (Xie et al., 2024). Nonetheless, its clinical translation remains limited by the pharmacokinetic challenges common to many small-molecule antioxidants, including poor bioavailability and inadequate tissue targeting.

In addition to its other mechanisms, berberine has been extensively investigated in the context of IVDD for its ability to activate the AMPK/Nrf2 signaling axis and inhibit the NLRP3 inflammasome. Berberine effectively attenuates extracellular matrix (ECM) degradation in intervertebral disc degeneration by suppressing the production of matrix-degrading enzymes. Furthermore, berberine treatment markedly activates autophagic flux in nucleus pulposus (NP) cells, and inhibition of autophagy significantly abrogates its protective effects against apoptosis and ECM catabolism, confirming that autophagy induction constitutes a key mechanism underlying its therapeutic action. In a rat needle-puncture model of IVDD, *in vivo* administration of berberine upregulates LC3 expression in disc cells and substantially delays the progression of degeneration (Chen et al., 2018). Collectively, these findings indicate that berberine exerts protective effects against NP cell apoptosis and ECM catabolism primarily through the induction of protective autophagy, thereby demonstrating clear therapeutic potential for the treatment of IVDD.

### 3.4 Curcumin

Curcumin has attracted considerable interest because of its potent anti-inflammatory and antioxidant properties, positioning it as a strong candidate for therapeutic intervention. *In vitro* studies show that curcumin effectively suppresses the expression of pro-inflammatory cytokines, particularly TNF-α and IL-6, while simultaneously stimulating the synthesis of type II collagen and proteoglycans. Similarly, galangin shows strong potential to halt IVDD progression through strategic modulation of the Nrf2/NF-κB signaling axis, offering a robust mechanism to counteract both inflammation and oxidative stress (Chen et al., 2025a). However, the clinical utility of free curcumin is severely hampered by its precarious physicochemical properties and poor pharmacokinetic profile. Its extreme hydrophobicity and negligible aqueous solubility lead to minimal intestinal absorption, while its inherent sensitivity to light and rapid metabolic degradation further shorten its biological half-life (Hao et al., 2025). Consequently, systemic administration fails to deliver effective therapeutic concentrations to the avascular and hostile disc microenvironment (Wang et al., 2023c). These profound delivery challenges have necessitated the development of advanced encapsulation strategies, such as the use of solid lipid nanoparticles and hydrogel scaffolds, to enhance curcumin’s stability and local bioavailability—a paradigm shift that will be discussed in detail in the following chapters.

### 3.5 Ozone therapy

Ozone (O_2_–O_3_) therapy is a minimally invasive clinical approach valued for its potent antioxidant and anti-inflammatory effects. Research by Elmounedi shows that it effectively slows early-stage IVDD progression by neutralizing oxidative stress and inhibiting the PI3K/Akt/NF-κB signaling pathway. Through this modulation of key intracellular signals, ozone therapy curbs the inflammatory cascade, helping to preserve disc structural integrity and offering a viable non-surgical option for managing initial degenerative changes (Elmounedi et al., 2024b). However, the study emphasized that ozone therapy is effective only in early disease stages and cannot reverse advanced inflammatory damage. In a clinical study by Sezen, intradiscal ozone therapy alone was compared with ozone combined with platelet-rich plasma (PRP) therapy (Sezen et al., 2025). Both regimens led to significant improvements in pain and disability scores with minimal side effects, yet no statistically significant difference emerged between the two groups. This result highlights the limitations of conventional single-modality approaches in addressing complex or advanced IVDD pathology. Because such strategies typically act through a single mechanism, they often fail to deliver the comprehensive intervention needed to counteract the multifactorial nature of disc degeneration. These shortcomings have spurred the development of advanced drug delivery systems and multifunctional biomaterials. Designed to overcome the constraints of traditional antioxidant therapies, these innovations enable more precise, efficient, and sustained treatment of IVDD. By serving as localized therapeutic reservoirs, they ensure targeted delivery and maintain the long-term stability required to effectively modulate the challenging microenvironment of the degenerating disc.

## 4 Recent advances in antioxidant therapy using biomaterials

Recent years have seen significant advances in the use of biomaterials for antioxidant therapy in IVDD. These innovations directly address the major limitations of traditional systemic drug administration, namely, low efficiency, poor targeting, and the associated risk of systemic side effects. By enabling targeted delivery and sustained release within a biocompatible framework, biomaterials markedly enhance the local concentration and retention of antioxidants in the degenerated disc. This localized strategy, which integrates multiple therapeutic functions, optimizes ROS clearance and thereby mitigates oxidative stress, suppresses inflammation, and slows extracellular matrix degradation. As a result, current research is shifting from simple, single-function systems toward more sophisticated, intelligent platforms that deliver integrated multifunctional capabilities (Hu B. et al., 2024; Shi S. et al., 2024; Song et al., 2024). This review summarizes recent advances in antioxidant biomaterial strategies—spanning hydrogels, nanomaterials, and hybrid systems that integrate cell- or gene-based therapies—organized by material type and therapeutic function, with an emphasis on their defining characteristics. Through deeper integration of materials science, biology, and clinical medicine, biomaterial-based antioxidant strategies offer considerable promise for developing more precise and effective treatments for IVDD.

### 4.1 Hydrogels as antioxidant carriers

Hydrogels have become highly promising platforms for regenerative therapy in IVDD owing to their distinctive structural features. Their high water content and excellent biocompatibility enable them to closely mimic the physicochemical properties of the native ECM, creating an ideal microenvironment for cellular repair. Additionally, their injectability supports minimally invasive delivery, allowing precise placement to restore disc integrity without extensive surgery (Yan et al., 2021; Li W. et al., 2023; Desai et al., 2024; Shan et al., 2025). However, conventional hydrogels, although they integrate well biologically, often exhibit limited load-bearing capacity and insufficient mechanical durability under the high-pressure conditions of the spine, rendering them suboptimal for applications requiring substantial structural reinforcement of the intervertebral disc (Mai et al., 2025). While emerging high-strength hydrogel formulations seek to address this limitation, achieving a balance between robust mechanical support and favorable biological integration remains an active area of investigation. Incorporating antioxidants into hydrogel systems enables localized, sustained, and targeted delivery, effectively scavenging ROS and promoting repair of degenerated intervertebral discs.

#### 4.1.1 ROS-responsive hydrogels

ROS-responsive hydrogels are engineered to detect and respond to elevated ROS levels in the degenerative intervertebral disc microenvironment. Upon sensing these biochemical cues, they either release antioxidants in a targeted manner or directly scavenge ROS to restore cellular homeostasis. Chen et al. developed an injectable photothermal hydrogel, Mn_3_O_4_@ChS-HA, that incorporates Mn_3_O_4_ nanoparticles to scavenge ROS and modulate ECM metabolism. Both *in vitro* and *in vivo* studies demonstrate that this hydrogel effectively inhibits apoptosis, delays cellular senescence, reduces inflammation, and promotes autophagy. When combined with mild photothermal therapy, the system further accelerates tissue regeneration and significantly slows IVDD progression (Chen et al., 2025e). Conley introduced a dynamic nano-hybrid peptide hydrogel (NHPH) capable of self-assembly. This material eliminates pro-inflammatory ROS, enhances ECM remodeling, and delivers pro-regenerative cytokines, thereby improving recovery after IVD injury (Conley et al., 2023). In another study, Gao et al. designed a ROS-responsive injectable hydrogel (PVA-tsPBA@SLC7A11 modRNA) for rapid loading and selective release of SLC7A11 modRNA. The system releases its payload in proportion to IVDD severity, inhibiting ferroptosis in NPCs and showing promise for early-stage IVDD treatment (Gao et al., 2024). Liu et al. engineered an injectable, inflammation-responsive hydrogel composed of methacrylated gelatin modified with phenylboronic acid groups and crosslinked with oxidized hyaluronic acid. This platform achieves high drug-loading capacity and enables controlled release triggered by both pH changes and ROS levels. In addition to its delivery function, the hydrogel exhibits intrinsic radical-scavenging and antimicrobial activities. Together with its excellent biocompatibility, these properties synergistically mitigate IVDD progression (Liu L. et al., 2024). Bu et al. constructed a reductive chelation hydrogel (HA-NCSN/Cu) through the reaction of hyaluronic acid with Cu^2+^. The resulting material displays strong photothermal effects and ROS-scavenging capabilities that jointly suppress inflammatory responses in NPCs. Beyond its antioxidant action, it actively promotes ECM remodeling by activating the TGF-β/Smad signaling pathway. This molecular activation stimulates structural protein synthesis, facilitating effective restoration of disc integrity and functional recovery in animal models (Bu et al., 2025). Yang et al. reported a nucleobase-driven, self-gelling injectable adhesive hydrogel (HAT) by incorporating thymidine into hyaluronic acid chains. The material exhibits excellent injectability, tissue adhesion, and hydration capacity. When loaded with MnO_2_ nanoparticles to modulate oxidative stress and hypoxia, it effectively promotes repair of degenerative intervertebral discs (Yang et al., 2024).

#### 4.1.2 Polysaccharide-based hydrogels

Polysaccharides are highly promising for IVDD regeneration owing to their biocompatibility, biodegradability, and structural versatility. These natural polymers can be readily tailored to meet specific clinical requirements, providing a flexible platform for scaffolds that closely replicate the native disc microenvironment (Shan et al., 2025; Wang X. et al., 2025). Yu developed a fucoidan-loaded nanofiber scaffold (F-PECUU) that promotes AF repair by mitigating inflammation and oxidative stress (Yu et al., 2022). *In vitro* studies show that the scaffold effectively suppresses inflammatory gene expression, prevents ECM degradation, and enhances production of key collagens and proteoglycans. These results are corroborated by *in vivo* data demonstrating reduced COX-2 expression, increased ECM deposition, and preservation of both disc height and nucleus pulposus hydration. In a related advance, W. Li engineered a composite hydrogel from fucoidan and glucomannan methacrylate that drives IVD regeneration through mechanotransduction and macrophage immunomodulation (Li W. et al., 2023). This hydrogel supports nucleus pulposus cell proliferation, modulates inflammatory responses, and activates the CAV1-YAP mechanotransduction pathway. In animal models, it suppressed inflammation, promoted M2 macrophage polarization, and attenuated ECM degradation.

Parallel to the limitations of bone implants, a primary bottleneck for hydrogels in nucleus pulposus (NP) repair is the mechanical mismatch within the spinal column. Although polysaccharide-based hydrogels like GelMA or HA offer excellent bioactivity, they frequently lack the structural robustness required for the disc’s high-pressure environment. This mechanical vulnerability leads to material fatigue and fragmentation under cyclic physiological loading, which not only compromises scaffold integrity but also risks triggering secondary inflammation from degraded debris (Sun et al., 2026). Currently, the inability to achieve precise anatomical conformability while maintaining adequate fatigue resistance remains a critical challenge, preventing these materials from ensuring long-term functional stability in the harsh, avascular disc niche.

#### 4.1.3 Protein-based hydrogels

In addition to polysaccharide-based and stimuli-responsive systems, protein-based hydrogels, particularly those derived from collagen and gelatin, offer superior bioactivity for disc repair. Gelatin methacrylate (GelMA) hydrogels have become an ideal platform for drug and cell delivery, thanks to their tunable mechanical properties and excellent biocompatibility. Their structure can be precisely adjusted to mimic diverse tissue environments, creating a versatile matrix that supports cell attachment and enables stable release of therapeutic agents (Li P. et al., 2023). Wang incorporated curcumin-loaded solid lipid nanoparticles into GelMA to fabricate Cur-SLNs/GelMA composite scaffolds (Wang et al., 2023c). These scaffolds effectively promoted regeneration of degenerated intervertebral discs, suppressed inflammatory mediators, and helped restore ECM metabolic balance. In another approach, Shi used GelMA hydrogels to deliver mitochondrial microvesicles (mitoMVs) derived from mesenchymal stem cells (MSCs), significantly improving outcomes in IVDD treatment. This system enhances MSC survival in the harsh disc microenvironment by inhibiting pyroptosis and preserving mitochondrial integrity. By blocking these cell death pathways and maintaining metabolic stability, the platform ensures that transplanted cells remain viable and functional, overcoming a major barrier to successful cellular therapy in degenerated discs. Yang et al. loaded GelMA hydrogels with interleukin-10 (IL-10) and kartogenin (KGN) to simultaneously target inflammation (via IL-10-mediated M2 macrophage polarization) and promote resident NPC anabolism (via KGN-induced chondrogenic differentiation), thereby supporting nucleus pulposus tissue regeneration in IVDD (Yang S. et al., 2025).

In a related study, Chen et al. engineered a high-performance, multi-dynamic crosslinked hydrogel to enable spatiotemporal delivery of siRNA for integrated gene–cell therapy. By precisely controlling the timing and location of genetic material release, this system optimizes the local therapeutic environment and enhances the efficacy of combined regenerative treatments (Chen J. et al., 2023). It also reduces inflammatory mediator secretion, mitigates nucleus pulposus cell degeneration, alleviates inflammatory storms, and promotes disc regeneration.

#### 4.1.4 Other functional hydrogels

**FIGURE 3:**
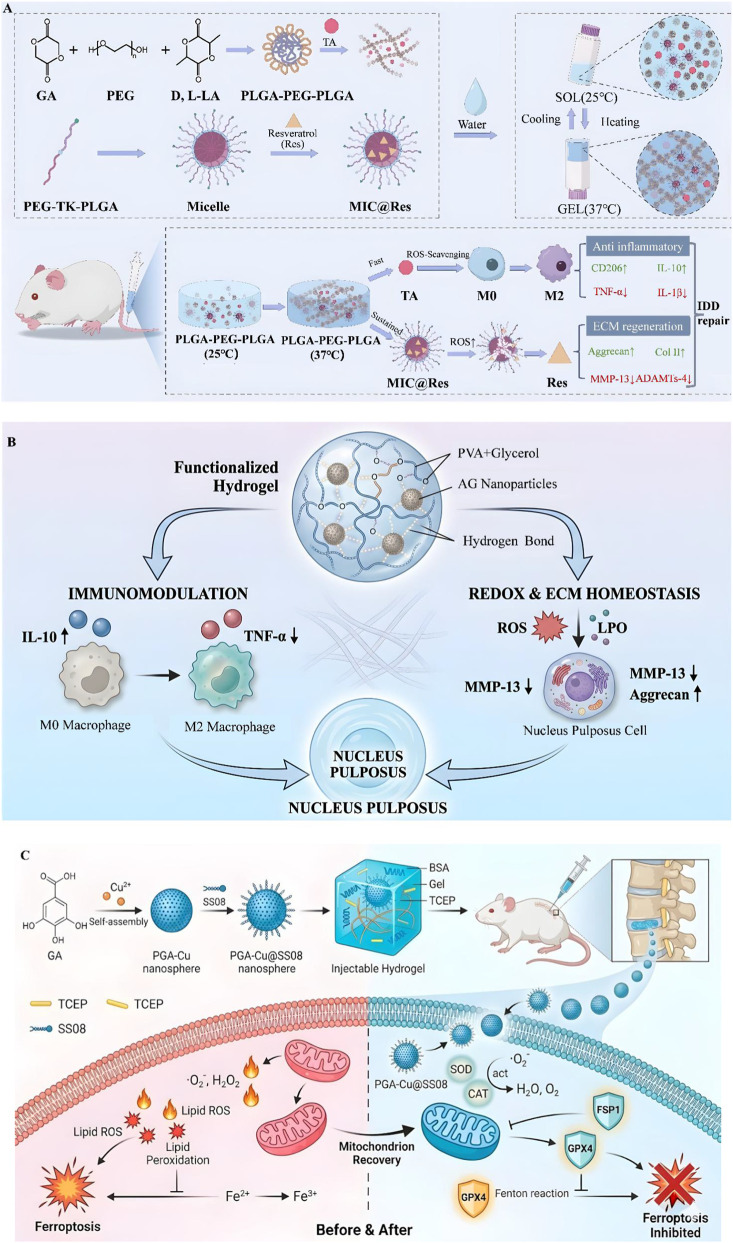
Application of injectable hydrogels in treating intervertebral disc degeneration. **(A)** Schematic illustration of the thermosensitive hydrogel drug delivery system design and associated mechanisms for repairing degenerated intervertebral discs (Liu et al., 2025). **(B)** Schematic of an injectable PVA/glycerol/AG hydrogel for IVDD repair via antioxidant, anti-inflammatory, and ECM-promoting mechanisms. **(C)** Guo et al. engineered a nanocomposite hydrogel embedded with mitochondria-targeting PGA–Cu nanoparticles functionalized with the SS08 peptide.

To address the limited efficacy of monotherapy in treating intervertebral disc degeneration, researchers have developed a series of functionalized hydrogels that enable multi-mechanism synergistic therapy. These systems leverage strategies such as microenvironment responsiveness, controlled release, and enhanced bioactivity to overcome the complexities of the disease. For instance, Chen et al. engineered an enzyme-initiated injectable hydrogel, composed of keratin methacrylate, that allows for the regulated release of exosomes. By suppressing inflammation and promoting the regeneration of the ECM, this platform provides a sophisticated approach to restoring disc health through coordinated biological pathways (Chen et al., 2024b). Zhou et al. developed an integrated synergistic therapeutic system (TMP@Alg-PBA/PVA) that combines ROS-responsive release, mitochondrial targeting, and concurrent antioxidant, anti-pyroptotic, and ECM-regenerative effects. In the high-ROS microenvironment of degenerative regions, the hydrogel triggers the release of tannic acid–manganese network nanoparticles (TMP NPs). These nanoparticles localize to mitochondria, where they efficiently scavenge ROS and mitigate oxidative stress at its source. Simultaneously, they inhibit the IL-17/ERK signaling pathway, thereby blocking NLRP3/Caspase-1/GSDMD-mediated pyroptosis in nucleus pulposus cells. The system also downregulates matrix metalloproteinase expression while stimulating type II collagen and proteoglycan synthesis, effectively delaying ECM degradation (Zhou et al., 2024a). Peng et al. reviewed the use of decellularized extracellular matrix (dECM) hydrogels in IVDD therapy, noting their capacity to improve mechanical properties, promote stem cell differentiation, restore disc height, and facilitate exosome release. Although studies show that dECM hydrogels can direct stem cells to differentiate into nucleus pulposus–or annulus fibrosus–like cells, universally accepted markers for these specific phenotypes remain undefined. Candidates such as KRT19 and GPC3 show promise but require further validation to confirm their reliability. Moreover, the precise mechanism by which dECM treatment ameliorates IVDD is still unclear; it likely involves a complex interplay of stem cell recruitment, dECM-mediated biomechanical cues, and multiple biochemical signaling pathways (Peng et al., 2023). In related research, Ma et al. developed a therapeutic system by combining a decellularized nucleus pulposus matrix (DNPM)/chitosan composite hydrogel with NPSCs and PLGA microspheres loaded with growth differentiation factor 5 (GDF5). The DNPM component retains the native ECM components and the intricate three-dimensional architecture of the nucleus pulposus, while chitosan enhances both mechanical stability and biocompatibility. Together, these elements create a synergistic microenvironment specifically optimized for NPSCs. The PLGA microspheres enable sustained GDF5 release, which continuously drives stem cell differentiation toward a nucleus pulposus–like phenotype, upregulating expression of type II collagen (COL2A1) and aggrecan (ACAN) and thereby restoring homeostasis of the nucleus pulposus matrix. The innovation of this approach lies in the targeted use of tissue-specific NPSCs, which exhibit higher differentiation efficiency than conventional bone marrow mesenchymal stem cells (BMSCs), combined with a strategic integration of a biocompatible scaffold, sustained-release growth factors, and specialized stem cells. This integrated platform enables precise repair and effectively reverses the pathological progression of IVDD (Ma et al., 2024). Additionally, X. Cheng and Wu developed a smart vanillin-crosslinked hybrid hydrogel for encapsulating IVDSCs, accelerating their differentiation and supporting regenerative repair of the degenerative nucleus pulposus (Cheng and Wu, 2024). Qing et al. designed programmable DNA hydrogels that enhance autophagy-mediated therapy for IVDD by establishing a favorable local microenvironment (Qingxin et al., 2023). This system employs spherical nucleic acids loaded with miR-5590, which the hydrogel delivers via localized, sustained release. By targeting and downregulating DEAD-box helicase 5 (DDX5) expression, miR-5590 inhibits mTOR phosphorylation and activates ULK1, thereby initiating protective autophagy in disc cells. This upregulation of autophagy serves a dual function: it suppresses apoptosis while simultaneously reducing levels of matrix-degrading enzymes, including MMP-3, MMP-13, and ADAMTS (Figure 3). As a result, the platform promotes synthesis of type II collagen and proteoglycans, facilitating reversal of degeneration and restoration of intervertebral disc structural integrity.

### 4.2 Nanomaterials and nanodrug delivery systems

Nanomaterials and nanodrug delivery systems (NDDSs) offer promising avenues for advancing antioxidant therapy in IVDD. Their unique physicochemical properties and high surface area enable controllable drug release and the ability to traverse complex biological barriers. These characteristics make nanotechnology particularly well suited for delivering therapeutic agents precisely to the challenging microenvironment of the degenerating disc (Elmounedi et al., 2024a; Hu Y. et al., 2024; Wang Y. et al., 2025). Nevertheless, clinical translation remains limited by concerns over the long-term biocompatibility and potential toxicity of nanomaterials (Li K. et al., 2024).

#### 4.2.1 Carbon dots/nanodots

Carbon dots and nanodots have emerged as a versatile platform for the development of antioxidant nanozymes due to their excellent biocompatibility and easy surface functionalization. Nanozymes are a specialized class of nanomaterials that mimic the catalytic activity of natural enzymes. By combining the stability of nanomaterials with the high catalytic efficiency of conventional enzymes, they effectively replicate endogenous antioxidant systems such as superoxide dismutase (SOD) and catalase (CAT). This dual functionality enables nanozymes to provide durable protection against oxidative damage within a robust synthetic framework (Jomova et al., 2024; Peng et al., 2025). In recent years, nanozyme therapy has emerged as a promising approach to counteract oxidative stress in intervertebral disc degeneration. Among the various platforms under investigation, carbon dot–based nanomaterials have drawn considerable interest because of their tunable antioxidant activity. These particles can be precisely engineered to mimic specific enzymatic functions, offering a stable and efficient means to neutralize ROS in the harsh disc microenvironment. W. Bu reported the development of glutathione-derived carbon dots (GSH-CDs) as novel antioxidant nanozymes (Bu et al., 2024). GSH-CDs mimic the cascade activities of multiple natural antioxidant enzymes, including SOD, CAT, and glutathione peroxidase (GPx), and efficiently scavenge excess ROS in nucleus pulposus cells. This action reverses oxidative stress–induced cellular senescence and restores matrix metabolism balance. By directly eliminating O_2_^−^, H_2_O_2_, and lipid peroxides, GSH-CDs restore mitochondrial membrane potential, suppress aging markers such as p21, and downregulate inflammatory and catabolic factors including TNF-α and MMP13. At the same time, they promote synthesis of key matrix components like aggrecan (Acan), ultimately reprogramming the nucleus pulposus microenvironment. Sun et al. developed manganese carbide nanodots (MCDs), a metal-based nanozyme with potent ROS-scavenging capacity (Sun et al., 2024). This material effectively inhibited necroptosis in nucleus pulposus cells, restored microenvironmental homeostasis, and demonstrated significant therapeutic efficacy in a rat model of disc degeneration. In another study, Zhang Qing found that selenomethionine-derived antioxidant carbon dots (Se-Met-CDs) exhibit potent antioxidant activity owing to selenium doping. By reducing intracellular ROS levels to just 17.7% of those in untreated controls, these nanodots effectively downregulate apoptosis-related proteins such as Caspase-3 and key inflammatory factors including TNF-α and IL-1β. The resulting suppression of oxidative stress activates the Nrf2 antioxidant pathway, which in turn upregulates synthesis of type II collagen (Col-II) and Acan. Simultaneously, the system inhibits matrix-degrading enzymes, specifically MMP-13 and ADAMTS-5, to prevent further tissue breakdown. Through these multidimensional mechanisms, Se-Met-CDs successfully restore nucleus pulposus cell function and maintain ECM homeostasis (Zhang Q. et al., 2025). However, current animal models rely exclusively on rat tail puncture to induce IVDD. This approach exhibits substantial anatomical and biomechanical differences compared with the human lumbar spine. Moreover, the short modeling period (8 weeks) fails to recapitulate the chronic, progressive nature of human IVDD, thereby limiting the clinical translatability of the reported therapeutic outcomes.

Compared with metal-based nanozymes, carbon-based nanomaterials generally exhibit higher biocompatibility and are more readily degraded and cleared from the body. However, their intrinsic antioxidant capacity is typically weaker than that of certain highly efficient metallic nanozymes, often necessitating surface modification or doping to enhance functionality. For example, in the study by Shi et al., carbon dots primarily serve as carriers and targeting ligands, with their intrinsic antioxidant activity playing a secondary role (Shi Y. et al., 2024). When designing carbon-based nanomaterials, it is essential to carefully balance their roles, as either functional therapeutic agents or functional delivery carriers, based on specific therapeutic requirements to achieve optimal treatment outcomes.

#### 4.2.2 Metal/metal oxide nanoparticles

In recent years, metal and metal oxide nanoparticles have emerged as a distinct and promising class of antioxidant materials for intervening in IVDD. Among these, nanoparticles possessing multi-enzyme activity and organelle-targeting capabilities have shown considerable potential. For example, H. Zhou’s team developed multifunctional PG@Cu nanoparticles with enhanced mitochondrial targeting and ROS scavenging ability (Zhou et al., 2024b). By inhibiting Gasdermin-mediated pore formation and blocking inflammasome activation, these nanoparticles effectively suppress necroptosis, thereby providing robust protection to the intervertebral disc. Their primary advantage lies in their capacity to precisely target organelles and modulate specific cell death pathways. In a complementary approach, Wang et al. engineered a core-shell nanozyme, designated PDA@CNO, consisting of a cobalt-doped NiO (CNO) core encapsulated within a polydopamine (PDA) shell. This architecture leverages the synergistic properties of both components to enhance therapeutic stability and catalytic performanc (Wang J. et al., 2024). Through the combined action of the Ni^3+^/Ni^2+^ and Co^3+^/Co^2+^ redox couples in the CNO core and the proton-coupled electron transfer capability of the PDA shell, the nanozyme mimics multiple antioxidant enzymes, including SOD and CAT, and efficiently scavenges ·O_2_^−^, H_2_O_2_, and ·OH. As a result, the system alleviates oxidative stress, suppresses inflammatory responses, protects nucleus pulposus cells, and promotes restoration of ECM homeostasis. Although this strategy has demonstrated significant therapeutic efficacy in early-stage IVDD rat models, owing to its structural design that enables multi-enzyme mimicry and enhanced electron transfer, certain limitations remain. Specifically, the long-term *in vivo* safety and metabolic fate of these materials are not yet fully understood, and the absence of validation in large animal models indicates that further research is essential before clinical translation can be considered.

The clinical translation of nanomaterials, particularly metallic nanozymes, is fundamentally constrained by long-term biosafety risks (Gao et al., 2026). A fundamental mismatch exists between the chronic nature of degenerative diseases and the cumulative toxicity of inorganic carriers. Metallic nanozymes can bioaccumulate in vital organs or undergo slow ion release, potentially inducing long-term cytotoxic effects. Furthermore, the “protein corona” masks engineered ligands, triggering macrophage uptake and the Foreign Body Response (FBR), which leads to fibrous encapsulation within the disc (Liu et al., 2026). Ultimately, excessive scavenging of reactive oxygen species may disrupt “redox eustress” (i.e., beneficial oxidative stress required for normal signaling), posing substantial biosafety risks associated with biopersistence in long-term clinical scenarios.

#### 4.2.3 Metal-organic frameworks (MOFs)

By targeting multiple cell death and inflammatory pathways implicated in IVDD, nanocarrier-based delivery strategies offer a sophisticated platform for precise clinical intervention. A compelling example comes from Zhang and colleagues, who used high-throughput screening of an FDA-approved drug library to identify pirfenidone (PFD) as a potent inhibitor of pyroptosis in NPCs. To optimize delivery, they engineered a pH-responsive metal-organic framework (MOF) carrier composed of polyhistidine-zinc (PHZ) to encapsulate PFD, yielding a PFD@PHZ composite specifically tailored to the pathological environment of IVDD (Zhang Y. et al., 2025). This system exploits the acidic microenvironment of degenerated discs to trigger targeted PFD release; PFD inhibits NLRP3 inflammasome activation by forming hydrogen bonds with NLRP3, thereby blocking the Caspase-1/GSDMD-mediated pyroptosis pathway. It also downregulates senescence markers (p16, p21, p53) and matrix-degrading enzymes (MMP13, ADAMTS5), while upregulating type II collagen synthesis, collectively improving NPC function and restoring ECM homeostasis. However, the PHZ carrier primarily functions as a delivery and sustained-release vehicle and lacks intrinsic anti-inflammatory or antioxidant activity; its targeting efficiency also requires further improvement. In another study focused on gene regulation, Li et al. developed histidine-engineered ZIF-8 nanoparticles (H-ZIF-8) for efficient siRNA delivery and gene therapy in IVDD (Li S. et al., 2025). H-ZIF-8 nanoparticles significantly enhance siATF3 delivery to NPCs, where they effectively suppress ferroptosis and ECM degradation by inhibiting the ATF3-mediated signaling pathway, thereby alleviating disc degeneration symptoms *in vivo* through optimized genetic drug delivery and precise modulation of ferroptosis (Table 1). Despite these promising results, the long-term safety of the system, the specificity of gene silencing, and the stability of the carrier within the complex degenerative microenvironment require systematic evaluation. Collectively, these studies highlight the potential of functionalized nanocarriers for targeted treatment of IVDD by addressing distinct pathological mechanisms, specifically pyroptosis and ferroptosis, and underscore that integrating multifunctional properties with high delivery precision represents a critical direction for future carrier optimization.

**TABLE 1:** Comparative analysis of antioxidant materials for intervertebral disc degeneration treatment.

Category	Antioxidant biomaterials	Experimental subject	Mechanism	Biological effect	References
Nanoparticles	CeO_2_ nanoparticles, Drug-loaded antioxidant nanoparticles	Human nucleus pulposus tissue, SD rat IVDD model	Scavenge ROS, Inhibit ferroptosis, Repair mitochondrial function	Protect nucleus pulposus cells, Preserve disc function, Reduce matrix degradation	Wu T. et al. (2025)
Nanozymes	Glutathione-doped carbon dots	Rat NPCs, rat model	Multiple antioxidant enzyme activities, ROS scavenging	Improves mitochondrial function, inhibits cellular senescence and inflammation	Bu et al. (2024)
Nanozymes	Carbonized Mn-containing nanodots (MCDs)	Human NP cells, Rat model	ROS scavenging, Inhibiting NLRP3 pyroptosis	Mitochondrial protection, Pyroptosis inhibition, ECM homeostasis restoration	Sun et al. (2024)
Nanoenzyme	PEG-CeO_2_ NPs	Rat nucleus pulposus cells, Rat IVDD model	Scavenge ROS, Inhibit cell apoptosis, Delay cell senescence	Protect nucleus pulposus cells, Improve matrix metabolic balance, Reduce degeneration degree	Chang et al. (2025)
Nanocomplexe	Cerium-luteolin nanocomplexes (CeLutNCs)	Macrophage, Renal epithelial cells, AKI mouse model, ALI mouse model	Scavenge ROS,Regulate macrophage polarization, Inhibit NF-κB pathway	Alleviate oxidative stress, Relieve inflammation, Repair damaged tissues	Gu et al. (2024)
Nanoparticles	Curcumin	SD rats, Rat nucleus pulposus cells	Inhibiting NF-κB pathway, Reducing pro-inflammatory cytokines	Suppressing TNF-α and IL-6, Restoring Col II and aggrecan expression	Wang et al. (2023c)
Nanoparticles	PGA-Cu@SS08 nanoparticles	Rat nucleus pulposus cells, Rat IVDD model	Activate NRF2 pathway, Inhibit ferroptosis, Repair mitochondrial function	Protect nucleus pulposus cells, Stabilize extracellular matrix	Guo Z. et al. (2025)
Nanoparticles	EGCG-copper ion metal-phenolic network	Rat and human NPCs, SD rat disc puncture model	Mitochondrial targeting, Lysosomal escape, Inhibition of NLRP3 inflammasome and GSDMD cleavage	Preserves disc height and water content, Maintains tissue structure, Reduces apoptosis and pyroptosis	Zhou et al. (2024b)
Nanomaterial	MPB-Mn3-CB nanoparticles	Human nucleus pulposus cells, Rat IVDD model	Scavenge ROS, Inhibit P53 pathway, Promote ECM synthesis, Delay cellular senescence	Protect nucleus pulposus cells, Reduce degeneration degree	Zhang A. et al. (2025)
Nanoparticles	Pirfenidone	Rat NPCs, Rat disc puncture model	pH-responsive drug release, Inhibition of NLRP3 inflammasome and GSDMD cleavage	Inhibits pyroptosis, Inhibits senescence, Inhibits ECM degradation, Alleviates IVDD	Zhang Y. et al. (2025)
Nanomaterial	CD-PB-TPP nanoparticles	Rat nucleus pulposus cells, Rat IVDD model	Scavenge ROS, Target mitochondria, Repair mitochondrial function, Inhibit cellular senescence	Protect nucleus pulposus cells, Improve matrix metabolism, Reduce degeneration degree	Shi Y. et al. (2024)

#### 4.2.4 Biomimetic nanoparticles

In recent years, cell membrane-camouflaged nanoparticles have demonstrated significant multitarget therapeutic potential for treating IVDD. A notable example is the biomimetic nanoparticle (TMNP@SR) developed by K. Li et al., which consists of a rapamycin-loaded mesoporous silica core encapsulated by an engineered macrophage membrane overexpressing TrkA. This system acts as an “inflammatory cytokine sponge” by simultaneously adsorbing inflammatory cytokines and nerve growth factor (NGF) while using homologous targeting to deliver rapamycin (RAPA) directly to macrophages. Upon exposure to the acidic disc microenvironment, RAPA is released in a sustained manner to trigger macrophage autophagy and drive polarization from the pro-inflammatory M1 phenotype toward the reparative M2 state. This phenotypic shift effectively suppresses inflammatory cascades, inhibits nucleus pulposus cell apoptosis, and prevents extracellular matrix degradation, as evidenced by upregulated COL2A1 and SOX9 expression alongside downregulated MMP3 and MMP13, while also alleviating neuropathic pain and neural infiltration (Li K. et al., 2024). The innovation of this approach lies in its integration of immunomodulation, anti-inflammatory activity, and pain relief, though further optimization is needed to improve the stability of macrophage polarization, assess long-term effects on nociceptive signaling, and enhance targeting efficiency. In contrast, Wang et al. reported a stem cell membrane-camouflaged ZIF-8 nanoparticle system (M@Z/S) that efficiently delivers shRNA to silence the LEPR gene, thereby inhibiting leptin pathway activity, reducing disc inflammation, decreasing expression of matrix-degrading enzymes, and promoting synthesis of extracellular matrix components (Wang Z. et al., 2024). The efficacy of this system stems largely from the stem cell membrane coating, which confers excellent biocompatibility, prolongs systemic circulation, and enhances both cellular targeting and uptake to enable efficient, sustained release of genetic therapeutics. Despite these advantages, clinical translation remains hindered by several key challenges, including the need for standardized, large-scale production of stem cell membranes and comprehensive *in vivo* evaluation of long-term safety and immunogenicity. Moreover, the stability of the targeting mechanism and the durability of gene silencing must be rigorously validated within the complex and hostile microenvironment of the degenerated human disc.

### 4.3 Antioxidant strategies in cell and gene therapies

The integration of antioxidant approaches with cellular or gene therapies represents a significant paradigm shift in the treatment of intervertebral disc degeneration, targeting its multifactorial pathology at the source to promote functional regeneration. Central to this synergistic strategy is the use of advanced biomaterials as multifunctional platforms that overcome the limitations of single-modality therapies. Antioxidants not only directly neutralize excess reactive oxygen species in degenerative discs but also, critically, create a protective microenvironment that shields transplanted therapeutic cells, such as mesenchymal stem cells, and resident cells from oxidative damage. This dual action markedly enhances cell survival, retention, and reparative function within the disc. At the same time, gene therapy delivered via biomaterial carriers enables precise upregulation of endogenous antioxidant defenses. By supporting sustained, autonomous protection, this system empowers disc cells to maintain their own redox balance and effectively restore overall tissue homeostasis.

#### 4.3.1 Cell therapy

Mesenchymal stem cells (MSCs) have attracted considerable interest owing to their ability to differentiate into multiple lineages, modulate immune responses, and exert beneficial paracrine effects. Despite this promise, their survival and functional efficacy are frequently compromised by the hostile microenvironment of degenerative intervertebral discs. To address this limitation, Shi and colleagues demonstrated that static magnetic fields effectively stimulate MSCs to release mitoMVs, thereby enhancing therapeutic impact through externally controlled physical stimuli that trigger secretion of specialized restorative factors (Shi P. et al., 2024). These mitoMVs reduce senescence in NPCs and, when delivered via a GelMA hydrogel system, slow IVDD progression. In another study, Fu reported that BMSCs suppress oxidative stress-induced ferroptosis in annular fibrosus cells (AFCs) by inhibiting STAT3 activation, and that implantation of BMSCs encapsulated in a matrix hydrogel mitigates IVDD development (Fu et al., 2025). Huang found that encapsulating MSCs in single-cell microgels improves their therapeutic efficacy against IVDD (Huang et al., 2024) by preserving mitochondrial function to inhibit pyroptosis and support MSC survival in the adverse disc environment. Y. Yang designed NPC-loaded hydrogel microspheres inspired by the “seed–soil” concept (Yang Y. et al., 2025), which integrate antioxidant and cell therapy functions into a unified strategy for IVDD treatment. Chen proposed a dual-pathway cascade approach using engineered apoptotic vesicles derived from MSCs (Chen et al., 2025d) that, when modified with an MMP13-responsive cell-penetrating peptide, target cellular senescence and hold therapeutic potential for IVDD. J. Zhao performed a meta-analysis of MSC-based IVDD therapies published between 2000 and 2024 (Zhao et al., 2025), concluding that future work must focus on enhancing MSC functionality, advancing cell-free therapies, and elucidating underlying molecular mechanisms.

Exosomes and extracellular vesicles (EVs) serve as key mediators of intercellular communication. They carry a diverse array of bioactive molecules, including proteins, lipids, mRNA, and miRNA, and hold significant promise for tissue repair and anti-inflammatory applications (Wang H. S. et al., 2024; Bhat et al., 2025; Chen et al., 2025b). W. Zhang developed self-powered triboelectric responsive microneedles capable of enabling controlled release of optogenetically engineered EVs for the treatment of IVDD (Zhang W. et al., 2024). This system restored TREX1-mediated clearance by delivering the TRAM1 protein, thereby counteracting cytoplasmic DNA sensing triggered by excessive mechanical stress. K. Zhang demonstrated that small extracellular vesicles derived from M2 macrophages (M2-sEVs) alleviate pyroptosis and IVDD through miR-221-3p–mediated suppression of both PTEN and NLRP3 (Zhang K. et al., 2024). In a separate study, Chen reported that exosomes derived from human bone marrow mesenchymal stem cells (hBMSCs), which carry U2AF2, effectively mitigate IVDD. This therapeutic effect is achieved by specifically modulating the circ_0036763/miR-583/ACAN signaling axis, thereby preserving disc structural integrity (Chen et al., 2024d). Using single-cell sequencing, Koroth identified a novel macrophage subset induced by MSCs (Koroth et al., 2023b). The study revealed that under hypoxic conditions, MSCs can reprogram macrophage phenotypes via TGF-β–dependent mechanisms, a finding with important implications for IVDD therapy.

Moreover, preclinical animal studies and early-phase clinical trials conducted by Chung indicate that enriched peripheral blood mononuclear cells (PBMCs) may offer a viable therapeutic approach for IVDD (Chung et al., 2024). These cells demonstrated potential in alleviating pain and enhancing quality of life. C. Chen further showed that allogeneic fibroblasts ameliorate IVDD in a rabbit model by reducing osteophyte formation (Chen et al., 2024a). Finally, Tamagawa provided a comprehensive review of recent advances in cell-based therapies for intervertebral disc regeneration and highlighted key barriers to clinical translation, particularly those concerning safety and efficacy (Tamagawa et al., 2024).

#### 4.3.2 Gene therapy

Gene therapy delivers therapeutic genes into biomaterials to enable localized and sustained gene expression, thereby regulating the biological activities of intervertebral disc cells. For example, A. Li developed surface-bound, gene-functionalized injectable short fibers (p16-LP@SF) that slow IVDD by reversing collagen aging (Li A. et al., 2025). These fibers release p16-siRNA in response to ROS, effectively mitigating NPC senescence while simultaneously enhancing collagen II synthesis and ECM remodeling. In a similarly strategic approach to IVDD treatment, S. Li used histidine-engineered ZIF-8 nanoparticles to facilitate siRNA delivery. This targeted intervention successfully inhibits ferroptosis and curtails ECM degradation, offering a promising pathway for alleviating disease progression (Li S. et al., 2025). In a review of recent advances, X. Li summarized progress in genetically functionalized regenerative materials for IVDD repair and emphasized their potential to overcome the limitations of conventional regenerative strategies (Li X. et al., 2024). In another study, G. Yang used single-cell RNA sequencing to guide mitochondrial therapy (Yang G. et al., 2025). The research team engineered exogenous mitochondria endowed with both antioxidant and anti-inflammatory functions. By modulating the mtDNA/SPARC-STING signaling pathway, this approach significantly improved therapeutic outcomes in IVDD.

**FIGURE 4:**
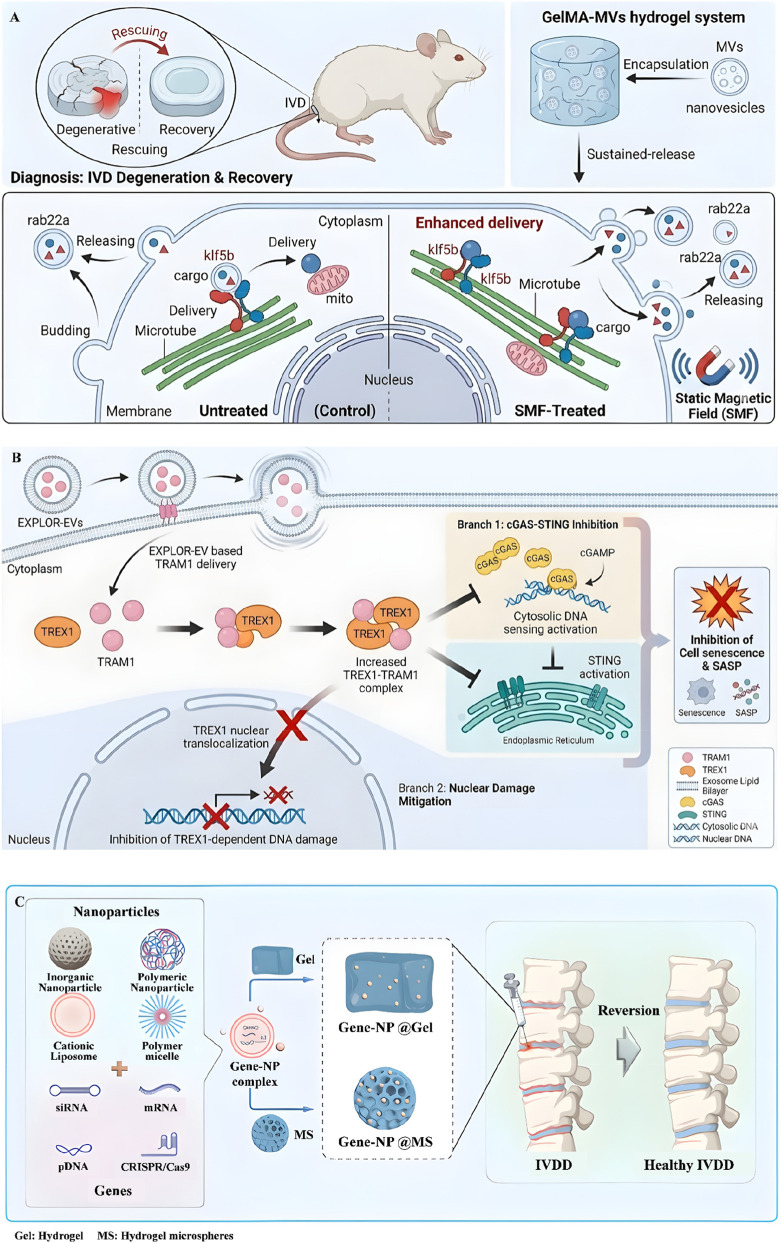
Antioxidant strategies in cell and gene therapies. **(A)** mitoMVs isolated following static magnetic field (SMF) intervention demonstrate significant therapeutic efficacy against IVDD when encapsulated in hydrogels. **(B)** Extracellular vesicle-delivered TRAM1 protein anchors TREX1 in the endoplasmic reticulum to clear damaged deoxyribonucleic acid and suppress inflammation, thereby alleviating IVDD. **(C)** Schematic illustration of the preparation of GRM and its application in disc repair (Li X. et al., 2024). Gel: Hydrogel. MS: Hydrogel microspheres.

MicroRNAs (miRNAs) also play a critical role in the pathophysiology of IVDD. Nanomedicines targeting miRNAs represent a promising strategy for regulating gene expression in IVDD treatment. Genedy and colleagues systematically reviewed the association between miRNAs and IVDD, underscoring the therapeutic potential of miRNA-based nanomedicines (Genedy et al., 2024). For instance, Jiang and coworkers delivered miR-150-5p using nanoparticles functionalized with an NPC-specific aptamer (Jiang et al., 2024). This method effectively reduced NPC senescence *in vitro* and slowed IVDD progression *in vivo*, demonstrating considerable therapeutic promise. The underlying mechanism involves targeting FBXW11 to inhibit TAK1 ubiquitination, thereby downregulating NF-κB signaling (Figure 4). This precise suppression of inflammatory pathways, mediated through specific molecular interactions, drives the observed regenerative effects.

### 4.4 Multifunctional composite materials and smart response systems

To address the complex pathological landscape of IVDD, current research focuses on developing composite materials and smart response systems that integrate multiple therapeutic functions. These advanced platforms are engineered to overcome the unique challenges of the disc microenvironment and enable a multifaceted treatment strategy by combining environmental sensing with targeted delivery. This shift toward intelligent, integrated biomaterials represents a significant advancement in managing the multifactorial nature of spinal degeneration. Specifically, these systems detect pathological cues in the disc, such as elevated ROS levels, pH shifts, and inflammatory mediators, and respond by releasing therapeutics or activating treatment modalities on demand, thereby enabling more precise and efficient IVDD therapy.

#### 4.4.1 Multifunctional composite materials

Yang et al. developed “seed-soil”-inspired hydrogel microspheres loaded with NPCs, which integrate antioxidant and cell therapy functions (Yang Y. et al., 2025). This approach establishes a new paradigm for IVDD treatment by combining the antioxidant properties of biomaterials with the regenerative capacity of cells, offering a promising strategy for synergistic therapeutic enhancement. Chen et al. designed a photothermal hydrogel, Mn_3_O_4_@ChS-HA, that scavenges ROS, modulates ECM metabolism, and enhances tissue regeneration through mild photothermal therapy, demonstrating the advantages of multifunctional synergy (Chen et al., 2025c). Guo et al. reported an ultrasound-responsive sericin/graphene oxide hydrogel loaded with rutin, engineered to target the Tie2-mediated, CBL-ubiquitinated EGFR axis. Upon ultrasound stimulation, localized rutin release elicits coordinated anti-inflammatory, pro-anabolic, and anti-catabolic effects, providing a multifunctional platform for IVDD therapy (Guo Y. et al., 2025). Zhou et al. created immune defense microspheres that combine cell membrane coating mimicry with surface chemical modification (Zhou L. et al., 2024). These microspheres promote nucleus pulposus regeneration by capturing and neutralizing inflammatory cascades, effectively restoring disc height and improving both the structural integrity and biomechanical function of the nucleus pulposus. Chen et al. proposed a dual-pathway cascade strategy using MMP13-responsive, cell-penetrating peptide-engineered mesenchymal stem cell-derived apoptotic vesicles to modulate cellular senescence, presenting a novel therapeutic avenue for IVDD (Chen et al., 2025d). In addition, Bu et al. constructed a reductive chelate hydrogel (HA-NCSN/Cu) that combines ROS scavenging, photothermal activity, and activation of the TGF-β/Smad pathway (Bu et al., 2025). By simultaneously alleviating oxidative stress and supporting disc matrix reconstruction, this dual-action system delivers a comprehensive therapeutic solution. The integration of protective and regenerative functions, critical for stabilizing the cellular microenvironment, enables effective disc self-repair under challenging physiological conditions.

#### 4.4.2 Smart responsive delivery systems

Stimulus-responsive delivery systems adapt effectively to the degenerative intervertebral disc microenvironment by responding to a range of internal and external triggers. Internal cues, such as pH fluctuations, reactive oxygen species (ROS) levels, and enzymatic activity, provide biological signals for drug release, while external stimuli like light, ultrasound, and magnetic fields enable precise physical control over therapeutic activation. This dynamic responsiveness bridges the gap between passive delivery and active intervention, allowing highly localized and timely treatment of disc pathology (Tang et al., 2024). By supporting targeted and sustained drug release, these systems maximize therapeutic efficacy while substantially minimizing systemic side effects. A notable example is the injectable anti-inflammatory hydrogel developed by Liu et al., which responds directly to pathological cues in the disc microenvironment to enable controlled release of diol-based drugs. This localized strategy maintains high drug concentrations at the injury site, ensuring both potent and biocompatible therapeutic action over an extended period. The system effectively alleviates IVDD while exhibiting excellent biocompatibility (Liu L. et al., 2024). Similarly, Zhou et al. designed a microenvironment-responsive metal–phenolic network delivery platform tailored to IL-1β-induced IVDD conditions. It exerts broad therapeutic effects by scavenging ROS, inhibiting necroptotic apoptosis, and actively promoting ECM regeneration (Zhou et al., 2024a). It exerts a broad range of therapeutic effects by scavenging ROS, inhibiting necrotic apoptosis, and actively promoting ECM regeneration. In a similar advancement, J. Chen and colleagues developed a high-performance hydrogel based on multi-dynamic bond crosslinking, which, through its unique structural properties, enables precise spatiotemporal delivery of siRNA. This transition from basic biochemical protection to sophisticated, time-controlled molecular therapy represents a significant leap in managing localized disc environments (Chen J. et al., 2023). By suppressing inflammatory mediators and protecting NPCs from degeneration, this system alleviates inflammatory storms and enhances intervertebral disc regeneration, representing a promising gene–cell combination therapy (Table 2).

**TABLE 2:** Comparative analysis of antioxidant materials in IVDD treatment.

Method	Core mechanism	Advantages	Disadvantages	References
Traditional antioxidants	Directly scavenge ROS, suppress inflammation	Readily available, Some low-cost	Poor bioavailability, Poor targeting, Short half-life, Single mechanism of action, Risk of systemic toxicity	Lu et al. (2023), Zhao et al. (2023), Zhu et al. (2023b)
Antioxidant hydrogel	Localised, sustained-release delivery of antioxidants, ECM mimicry, ROS responsiveness	Improved biocompatibility, injectable, Localised sustained release, Capable of mimicking ECM, partially ROS-responsive	Insufficient mechanical strength, Long-term stability, Safety of degradation products, Complex preparation process	Chen et al. (2025e), Conley et al. (2023), Wang et al. (2023c), Bu et al. (2025)
Antioxidant nanomaterials	High ROS scavenging efficiency (nanoenzymes)	High surface area, Enzyme-mimetic activity, Targeted delivery, Transcending biological barriers, Multifunctionality	Potential toxicity, Complex synthesis, Challenges in large-scale production, Insufficient stability	Bu et al. (2024), Sun et al. (2024), Wang J. et al. (2024), Zhou et al. (2024b)
Gene therapy	Enhances cell survival/function, Targets gene expression	Addresses pathological root causes, promotes tissue regeneration, delivers sustained therapeutic effects, synergises with antioxidants	Cell survival/homing, Immunogenicity, Gene delivery safety, Ethical concerns	Genedy et al. (2024), Shi P. et al. (2024), Fu et al. (2025), Li A. et al. (2025)
Smart response system	Integrates multiple therapeutic modalities, Responds to changes in the pathological microenvironment	Synergistic enhancement, Precision personalised treatment, On-demand drug release, adapts to dynamic environments	Complex design/preparation, Difficult to produce at scale, Challenges in clinical translation	Tang et al. (2024), Zhou et al. (2024a), Chen et al. (2025c), Yang Y. et al. (2025)

## 5 Prospects and challenges

Over the past decade, research on antioxidant therapies for IVDD has made considerable progress. Ongoing studies have significantly advanced our understanding of how OS contributes to IVDD pathophysiology, laying the foundation for innovative antioxidant materials and delivery systems that offer renewed hope for effective treatment. Despite these scientific advances, which bridge the gap between basic research and therapeutic potential, the translation of such promising findings into real-world clinical applications continues to face multiple, complex challenges.

Future developments in antioxidant materials are expected to advance along several fronts. Researchers are working to identify safer and more effective antioxidants from both natural and synthetic sources and to enhance their targeting precision and stability through structural modification and nanotechnology. Mechanistically, the focus is shifting beyond conventional free radical scavenging. Current efforts increasingly center on nanomaterials that mimic endogenous antioxidant enzymes, particularly superoxide dismutase, and on intelligent systems designed to activate the body’s intrinsic defense pathways. By harnessing these biomimetic and responsive technologies, scientists aim to develop more sophisticated therapeutic interventions that operate in concert with natural biological processes (Lu et al., 2023; Mao and Fan, 2024; Sun et al., 2024; Chen et al., 2025c). To overcome the pharmacokinetic limitations of natural products, researchers have increasingly turned to nanotechnology to develop advanced delivery systems that enhance targeting specificity and bioavailability. This approach represents a highly promising integrative strategy, effectively combining the inherent bioactivity of natural compounds with the superior delivery capabilities of nanomaterials. A notable example is the work of Gu et al., who developed a self-assembled nanocomplex from luteolin and cerium ions. This design not only addresses luteolin’s poor water solubility but also increases its local concentration at pathological sites by exploiting the enhanced permeability and retention (EPR) effect characteristic of nanocarriers (Gu et al., 2024). Beyond improving delivery, the cerium ions themselves possess enzyme-mimetic antioxidant activity, resulting in a synergistic effect in which the combined system outperforms either component alone. Another innovative strategy involves the indirect use of natural products, as demonstrated in a study by Peng et al. By pretreating MSCs with quercetin, the researchers enriched the cells’ secreted exosomes with more potent antioxidant and anti-inflammatory cargo (Peng et al., 2024). When delivered to NPCs, these “primed” exosomes more effectively suppressed NLRP3 inflammasome activation and pyroptosis. The key innovation lies not in direct drug delivery, but in using the natural compound to “train” cells to produce more therapeutically effective exosomes, essentially engineering natural nanomedicines. This paradigm opens a novel avenue for harnessing natural products in regenerative therapy. Compared with conventional drug-loaded nanoparticles, such exosome-based approaches may offer superior biocompatibility and reduced immunogenicity. Collectively, these studies highlight that integrating natural products with advanced nano-delivery systems is a critical step toward unlocking their full therapeutic potential and accelerating clinical translation.

Moreover, an ideal antioxidant approach should extend beyond passive defense to actively remodel the disc microenvironment. This can be accomplished by incorporating growth factors or gene regulatory elements that synergistically enhance ROS scavenging while promoting extracellular matrix synthesis and cellular regeneration, ultimately enabling true tissue repair (Li X. et al., 2024; Ma et al., 2024). Given the intimate coupling of biomechanics and biochemistry in the intervertebral disc, future studies must more thoroughly integrate biomechanical considerations to develop materials capable of both effectively neutralizing ROS and providing appropriate mechanical support.

The avascular nature of the intervertebral disc (IVD) creates a “delivery desert” for biomaterials. Key biophysical barriers include: (i) a dense, polyanionic extracellular matrix that impedes diffusion and traps charged carriers; (ii) the absence of systemic convection, restricting transport to slow diffusion; (iii) a hostile microenvironment (high osmotic/hydrostatic pressure and low pH) that destabilizes delivery systems; and (iv) sparse cellularity that limits intracellular therapeutic uptake (Blanquer et al., 2015; De Geer, 2018). This unique structural barrier renders conventional delivery methods, such as oral or systemic administration, largely ineffective for antioxidant therapy. Because achieving therapeutically relevant concentrations at the target site through these routes is extremely difficult, maintaining sustained release and long-term efficacy of antioxidants remains a major clinical challenge. Many biomaterials undergo rapid degradation or lose bioactivity over time, often necessitating repeated treatments or additional interventions, an approach that may be feasible in acute settings but is impractical for the chronic, long-term management of conditions like intervertebral disc degeneration (Mai et al., 2025). To address these limitations, researchers developed a biopolymer-based dynamic hydrogel (HPPC) that responds rapidly to the elevated ROS levels and acidic pH characteristic of the degenerative disc microenvironment. By enabling on-demand and sustained release of antioxidant drugs directly at the pathological site, this responsive system targets the core drivers of disease progression. Both *in vitro* and *in vivo* studies demonstrate that HPPC effectively alleviates oxidative stress and inflammation in nucleus pulposus cells, primarily through modulation of the COX2–PGE2–NFκB and TNFα–P38–MAPK signaling pathways. Ultimately, this intervention restores disc function in rat models, establishing the HPPC hydrogel as a promising strategy for improved management of intervertebral disc degeneration (Wang S. et al., 2025). The HPPC-based dynamic hydrogel represents a versatile platform whose intelligent drug delivery mechanism holds potential not only for intervertebral disc degeneration but also for other tissue-level disorders.

Moreover, the long-term immunological compatibility of antioxidant biomaterials within the intervertebral disc (IVD) remains insufficiently addressed. While many systems pass short-term biocompatibility screenings, the chronic foreign body response (FBR)—driven by macrophage recruitment and fibrotic encapsulation—can compromise both structural integrity and therapeutic efficacy. Lessons from spinal arthroplasty reveal that non-cytotoxic wear debris (e.g., metal, PEEK, or polyolefins) can trigger a sterile inflammatory cascade, characterized by the release of pro-inflammatory cytokines (IL-6, TNF-) and matrix metalloproteinases (MMPs) from resident disc cells and surrounding tissues (Werner et al., 2018; Wei and Zhu, 2025). Within the immunologically privileged environment of the NP, even inert nanoscale fragments may act as persistent inflammatory stimuli, potentially exacerbating disc degeneration rather than resolving it (Ortis et al., 2025).

While antioxidant-based materials represent a promising alternative for the treatment of intervertebral disc degeneration, their translation from the laboratory to clinical practice remains a lengthy and complex process. Currently, the majority of related research is confined to cellular and animal models, with multiple barriers impeding clinical application (van Uden et al., 2017; Xin et al., 2022; Tian et al., 2023; Tamagawa et al., 2024). First, the long-term biosafety of newly developed materials, particularly nanomaterials and smart responsive systems, requires further validation (Song et al., 2021; Yu et al., 2023). Key aspects such as their *in vivo* degradation behavior, potential toxicity, and capacity to elicit immune responses must undergo systematic evaluation (Wang J. et al., 2024). Beyond these preclinical findings, rigorous confirmation is still needed to ensure that these materials retain both stability and sustained antioxidant activity within the unique microenvironment of degenerated human intervertebral discs. From an industrial standpoint, additional challenges persist, especially regarding the complexity of manufacturing processes required to produce these materials while maintaining batch-to-batch consistency and achieving scalable production. When therapeutic strategies incorporate bioactive components, such as stem cells or exosomes, they must also meet more stringent ethical oversight and higher regulatory requirements. Addressing these fundamental issues is essential for antioxidant-based therapies to successfully advance from bench research to delivering tangible clinical benefits for patients (Zhao et al., 2025).

In summary, antioxidant materials hold significant potential for treating IVDD, offering the prospect of a paradigm shift in disease management. Realizing this vision, however, demands sustained global collaboration among scientists, engineers, and clinicians, coupled with continued investment in basic research, material innovation, and clinical standardization. Through these integrated efforts, which bridge the gap between laboratory discovery and patient care, more effective and safer therapeutic options can ultimately reach individuals suffering from IVDD.

## 6 Conclusion

IVDD is a prevalent and debilitating condition that has long posed a significant challenge in clinical practice. This review provides a comprehensive overview of recent advances in antioxidant materials, with a specific focus on the role of oxidative stress in driving IVDD pathology and on the potential of innovative materials and delivery systems to effectively counteract the degenerative process.

OS is a central driver of both the initiation and progression of IVDD, profoundly disrupting spinal tissue homeostasis. It promotes degeneration through multiple interconnected mechanisms, including the induction of NPC death, specifically via apoptosis, necroptosis, and ferroptosis, as well as the onset of cellular senescence. Beyond direct cell loss, oxidative stress triggers chronic inflammation, degrades the ECM, and impairs the essential functions of mitochondria and the endoplasmic reticulum (Wang et al., 2023b; Chen et al., 2024e; Zhou et al., 2024c). In response to this pivotal pathological process, conventional antioxidant strategies have been employed. However, their therapeutic impact has been limited by poor bioavailability, inadequate targeting, and short half-lives, all of which have hindered the achievement of satisfactory clinical outcomes (Kawabata et al., 2023; Chen et al., 2024e).

In recent years, biomaterials have significantly advanced antioxidant therapies for IVDD. Hydrogels, which are biocompatible and injectable, serve as carriers that mimic the natural ECM. By incorporating ROS-responsive elements, polysaccharide-based substances, or the GelMA platform, they enable localized, sustained, and targeted antioxidant delivery (Chen et al., 2025e; Yan et al., 2021; Conley et al., 2023; Desai et al., 2024). This approach effectively eliminates ROS and supports tissue regeneration. Leveraging their unique physicochemical properties and ability to cross biological barriers, nanomaterials and NDDSs offer highly precise and efficient antioxidant strategies. These platforms, including carbon dots, metal-based nanoparticles, MOFs, and biomimetic nanoparticles, combat oxidative stress through diverse mechanisms. By mimicking natural enzyme activity, directly scavenging ROS, or regulating specific gene expression, these systems provide a sophisticated approach to therapeutic intervention (Bu et al., 2024; Hu Y. et al., 2024; Sun et al., 2024; Zhou et al., 2024b).

Moreover, combining antioxidant approaches with cell and gene therapies represents a promising future direction for IVDD treatment. Strategies such as improving MSC survival and function, promoting intercellular communication via exosomes or EVs, and regulating gene expression using genetically functionalized materials or miRNA-based therapies may fundamentally reverse the IVDD process (Genedy et al., 2024; Shi P. et al., 2024; Fu et al., 2025; Yang Y. et al., 2025). By integrating diverse treatment modalities, multifunctional composites and smart responsive systems can intelligently adapt to the shifting pathological microenvironment of the disc. These advanced platforms, which actively sense and respond to localized biological changes, enable the delivery of precise, personalized therapy tailored to the specific needs of the patient (Chen J. et al., 2023; Tang et al., 2024; Zhou et al., 2024a).

While antioxidant materials show significant promise for treating IVDD, they continue to face substantial hurdles, chief among them the transition from laboratory research to clinical application. This translation requires rigorous, long-term assessment of safety, biocompatibility, and therapeutic efficacy. Moreover, there is an urgent need to refine personalized treatment strategies, standardize manufacturing protocols, and address complex ethical and regulatory challenges. Future work should prioritize elucidating the intricate molecular mechanisms of oxidative stress in the disc, potentially through multi-omics technologies to uncover novel therapeutic targets. Such efforts will drive the development of smarter, more efficient, and safer antioxidant materials and delivery systems. Concurrently, integrating biomechanical and biochemical considerations into the design of composite materials, so they provide both mechanical support and biological functionality, will be critical for achieving comprehensive regenerative therapy for IVDD.

In summary, advances in antioxidant materials for IVDD offer compelling potential to overcome the limitations of conventional therapies. Through sustained basic research and cross-disciplinary collaboration that bridges materials science and clinical medicine, these innovative strategies are poised for successful clinical translation. This progress promises more effective, durable treatment options for the large population of IVDD patients, substantially improving their quality of life.
